# Elderly Forgotten? Digital Exclusion in the Information Age and the Rising Grey Digital Divide

**DOI:** 10.1177/00469580221096272

**Published:** 2022-04-26

**Authors:** Farooq Mubarak, Reima Suomi

**Affiliations:** 1 Information Systems Science, 8058 University of Turku, Finland

**Keywords:** grey digital divide, information and communication technology, digital divide, digital exclusion

## Abstract

**Background:** Information and Communication Technology (ICT) is being spread at an unprecedented rate across the globe. Yet, new research suggests that digital divide is not only continuing but also deepening at the same time. After access to basic ICT equipment, it is now the lack of skills and quality of hardware and software that leads to a continuing digital divide. Digital divide which is specifically related to elderly is known as grey digital divide. **Objectives:** The focus of this paper is to review and analyze recent relevant research on grey digital divide which is fast emerging as a major challenge in the era of ageing. A side objective is to raise implications for theory and practice discourses to minimize grey digital divide. **Method:** Literature on digital divide and grey digital divide is reviewed to gather relevant knowledge on the grey digital divide. The articles were filtered according to the relevance for grey digital divide in the present age. Literature review spanned over a 5 years’ timeline to discuss the current trends on the topic. **Results:** Results indicate a rising problem of ageing, mainly in developed countries. Grey digital divide constitutes a major challenge for elderly to participate and benefit from the digital revolution. Elderly face problems for basic tasks such as booking tickets or renewing bus cards to claiming old-age benefits because most of the systems are digitized. Another challenge is the social exclusion faced by elderly because they cannot connect with peers through digital networks due to lack of digital skills. This situation is also observed in developing countries, although in developing countries elderly get immediate help from family members due to the family system of joint living. **Implications and future directions:** A practical implication of this research can be a full-scale fieldwork in different countries of the world to further understand the grey digital divide. Joint collaboration between ICT and healthcare industry may result in revolutionizing of ergonomic ICT products and services which are elderly-friendly. It would be interesting to know how culture impacts grey digital divide across various countries.

**What do we already know about this topic?**

The digital divide is becoming a significant research issue in today’s society.

**How does your research contribute to the field?**

It Fills a Research Gap by Creating a Scientific Understanding of the Grey Digital Divide

**What are your research’s implications towards theory, practice, or policy?**

A cross-collaboration among industrial, academic, and healthcare discourses to design innovative ICT-assisted solutions aimed at improving the welfare of senior citizens.

## Introduction

ICT (Information and Communication Technology) being an important component of digital ecosystem, is expanding its foothold worldwide at an unprecedented rate. At the same time, digital divide is reportedly deepening across the globe.^1,2^ A fresh example of this is provided by Sylvester, Toland and Parore^3^ who demonstrate marginalized communities deprived of digital technologies in New Zealand. Another example is afforded by Haenssgen^4^ in India where several marginalized communities continue to suffer from digital revolution despite mass diffusion of ICT in the country, with only rich being insulated from this divide. Admittedly, the digital exclusion is lower compared to the past; however, several remain excluded.^5^ Having solved access to basic ICT across major portion of the world, it is skills to use ICT which contributes to a continuing digital divide. For instance, equipment barrier, skills barrier, and health barrier are contributors to the digital divide, even when seemingly use of digital technology has increased during COVID-19.^6^ In addition, quality of hardware and software also affects one’s ability to use digital technologies which leads to a digital divide.

Studies over the past 2 decades have provided important information on the nature of the digital divide and various forms it takes. Up to now far too little attention has been paid to tracing a systematic solution to the socioeconomic problem of digital divide. In particular, digital divide is rising in the information age. For instance, ICT companies roll out new devices in the market each year, which creates a digital divide based on the quality of hardware. Similarly, new ICT content demands advanced skills which cannot be easily learnt by everyone. Hence, the digital divide is continuing. Take for example, the school system in developing countries which is often equipped with leftover and refuted technologies from the wealthy countries. Apparently, developing countries buy such stock in bulk and schools and other institutions purchase such items. Interestingly, this phenomenon is happening at the same time when ICT is being diffused at an increasing rate across the world (see for example,^7^).

Recently, in the era of ageing, another form of digital divide is actively emerging, particularly in the developed world. Admittedly, some research has pointed out digital divide related to elderly, a scientific understanding of how the grey digital divide is unfolding itself and how it can be controlled is still lacking. This indicates a need to understand the various perceptions of the grey digital divide among elderly and identify reasonable solution to this problem. Freedom of information is deemed as an important human right,^8^ and apparently grey digital divide denies this right to the elderly in the information age.

The focus of this paper is to review and analyze recent research on grey digital divide which is fast emerging as a major challenge in the era of ageing, particularly in the developed world. Digital landscape has changed considerable in the past few years, thus this paper shall particularly focus research studies taken place in the past few years. For the purpose of the present paper, focus is centralized on the research conducted in past 5 years on grey digital divide. A secondary aim is to shed light on the mental wellbeing of elderly with respect to the digital technology. Reader should bear in mind in mind mindthat it is not the goal of this paper to provide definitive solutions to the grey digital divide; instead it reflects on the underlying mechanisms governing the grey digital divide research.

## Method

The paper is designed to be exploratory and interpretive in nature. Literature on the digital divide and the grey digital divide is reviewed to gather relevant knowledge on the grey digital divide. Initial search through electronic databases revealed several references. A careful scan of literature brought forward the realization that research themes were highly diversified; and it was particularly difficult to find the research articles relevant to the aims of the present paper.

The articles were filtered according to the relevance for grey digital divide in the present age. Literature review spanned over a 5 year timeline to discuss current trends on the topic. Five year timeline was also selected due to the reason that most of the studies on grey digital divide emerged during this time-period. Since, the digital divide has been a contested theme across literature, the last 5-year timeframe gives us an opportunity to analyze the studies as the field gains maturity.

As the paper centers on the grey digital divide; earlier debates on digital divide in general are less essential to this research. However, a few references outside this timeline were also found to be relevant and could not be ignored with respect to the focus of the present paper. Articles were scanned for being relevant or not relevant. The inclusion criteria was selecting the articles which focus on elderly with respect to the disparities in using the digital technologies.

A double-check was performed to select the articles most-relevant to the goals of the current paper. Nevertheless, some interesting articles could not be added to the present study, in-order to remain within the scope of the research. This is a common issue with targeted literature reviews, and it is advisable that caution is exercised when interpreting the results.

## Literature Review

European commission warns that most of the world continents are forecast to witness a considerable ageing of their populations during 2015-2017.^9^ The report further adds that dependency ratios during this era are expected to increase significantly. Elderly continue to be digitally-excluded in the current information age,^10,11^ despite the mass diffusion of ICT across the planet. Choi et al.^12^ arrived at the conclusion that ageing has a strong association with decline in use of Internet. During COVID-19 crises, many elderly have experienced limited access to the specialist care.^13^; the results would have been different if elderly were digitally-skilled and if the digital healthcare infrastructure aimed at elderly was made available.

At the other end of the spectrum, digital technologies hold tremendous potential and promise to update the lives of elderly with respect to quality of living. For instance, in recent research, Francis et al.^14^ found that when elderly use digital technologies, they enhance their quality of life. For example, elderly can book the medical appointments over the internet, can manage the regular follow-ups with the doctors digitally, and report their medical conditions to their care-providers in a digital format. It is estimated that nearly sixty percent of the Internet users search health-related information online.^15^ This trend, however, may appear different across different countries. Many services are moving towards digital platforms including banking, pension, old-age benefits, TV services, transportation booking etc. Elderly face challenges to use many basic services just because they are unfamiliar with the use of digital technologies. At times, admittedly, elderly out of their own will w distance themselves with the digital technologies. Other times, they are unable to learn the digital skills on their own and require coaching which also is a tough call. This is because custom-tailored ICT-tuition programs as well as skilled tutors are required to work with elderly; this is not always easy to arrange.

There is a consensus among social scientists that the grey digital divide is getting serious with the passage of time.^16-18^ The most serious consequences of the grey digital divide are perhaps related to the healthcare as Fox and Connolly^16^ correctly indicate that elderly are unable to benefit from mobile health services despite the available access to ICT. Continuing in the same vein, Bercovitz and Pagnini^19^ inspire that digital technologies hold a tremendous potential for improving quality of healthcare of senior citizens.

The role of peer support especially family is vital in helping senior citizens to embrace ICT and benefit from it. For instance, Schreurs et al.^18^ maintain that family can vastly help elderly to participate in information society. Hong and Cho^20^ feel that health-related Internet use of senior citizens can be inspired by elderly-friendly online services that are easy to use.

However, a key challenge emerging fast in the digital society is the reliability of information of the Internet. Pálsdóttir^21^ performed an interesting analysis tracing the reliability of health information across media from 2002 to 2012 and found that while information seeking during this time period has increased, participants had also become critical of the information available with the passage of time.

This issue of reliable internet content is a challenge parallel to the accumulation of vast information on Internet each millisecond, often known as big data^22-24^ which makes it difficult to distinguish relevant information from irrelevant information. Although, elderly are skilled at making evaluations better than their younger-counter parts; it may still be frustrating to filter-out irrelevant or misleading information from the relevant. Similarly, health challenges that come with the old age may also add to the frustration of elderly while trying to engage with ICT. It is therefore understandable that digital exclusion of elderly does not always mean that they have themselves decided to refrain from digital inclusion (for instance consider,^25^).

The digital divide is a multifaceted phenomenon and needs multifaceted efforts to control it.^26-29^ In the same sense, the grey digital divide has to be tackled from multiple perspectives. It can no longer be assumed that providing access, skills and motivation to elderly will close the digital divide. This view is also echoed in the work of Nguyen et al.^15^ who inspire that tackling grey digital divide would involve input from multiple stakeholders including patients, medical practitioners, and ICT industry. Similar to the conclusions of Hakkarainen,^30^ Borg et al.^31^ also remind us that the grey digital divide is influenced not only by access but a myriad of factors including ICT skills, ability and social support.

Elderly, especially in the developed countries presumably have the resources to access ICT; benefitting from ICT is entirely another thing. For instance, a study performed on UK elderly clearly indicates a remarkable grey digital divide.^6^ Accurate training to elderly is of utmost importance and single most important factor of bringing them in the sphere of digital inclusion. Similarly, Mubarak and Nycyk^32^ propose highly-skilled training programs aimed at elderly to enable them in benefitting from ICT ecosystem. It also includes creating mindset in addition to delivering ICT skills to elderly. For example, one study^33^ after analyzing focus-group interviews with 30 elderly prospects aged 66-89 shows how they discursively create a divide between themselves and other ICT users.

Haenssgen^4^ shows how in future healthcare industry will increasingly adhere to mobile phone users in India but at the same time several communities will be pushed to digital exclusion. Healthcare may be a good motivation for the elderly to use ICT, as Haenssgen^4^ believed that the healthcare industry will target ICT users in future. The grey digital divide is now creating a health divide and social exclusion which should be alarming concern for researchers and practitioners. Social inclusion has been linked to health and wellbeing of elderly (34 [as cited by Careny & Kandt, 2022])

Such trend is already happening elsewhere in the world where mobile health solutions are increasingly being developed. Careny and Kandt^6^ argue that social inclusion of elderly can be achieved via engagement with digital technologies. A recent challenge regarding empirical research on mental-health related to elderly concerns with the social distancing guidelines with respect to the COVID-19 health emergency (as was also noted by,^13^). It was already proved that the digitally-excluded young adolescents tend to show deterioration in mental health than their digitally-included fellows^35^; this may be no different in the case of elderly, although a controlled-trial(s) is needed to confirm this.

Role of media in creating awareness about digital inclusion for elderly is undeniably significant, as Givskov and Petersen^36^ inspire that traditional society is currently undergoing a phase of mediatization. Video prompting mode of instruction aimed at elderly appears to be effective in bringing the elderly to digital inclusion.^37^ Media can be seen more as instrument of mass influence and holds the promise for influencing elderly decisions to embrace ICT. When coupled with customized training programs aimed at elderly, it is likely that the grey digital divide shall be controlled to a notable extent.

## Results and Discussion

The academic literature on the grey digital divide has revealed the emergence of several contrasting themes. Most researchers examining grey digital divide have utilized qualitative research. As mentioned in the literature review, grey digital divide is actively emerging as a new variant of digital divide. Seemingly, this belief among researchers is gaining popularity; digital divide exists even in the current information age. During the ensuing decade, the digital divide has been contested heavily among researchers with some arguing that it was over while others advocated a deepening digital divide. Earlier, it was hypothesized, although never really proven that the issue of digital divide solved itself. The overall consensus in the literature is that the grey digital divide is becoming increasingly significant area of research and policy concern in the information age. The problem is further compounded by the fast ageing across the globe. The concern over rising ratios of dependency burden of elderly expressed by European Commission multiple times in their annual reports is a neat example of why ageing comes with challenges for economy.

If incorporated effectively, the digital inclusion of elderly can help shun reliance on any option of specialized support concerning basic tasks. Although, ageing is progressively being seen as a weakness for the economy, it can arguably be the significant strength of a nation given their life experience, if the elderly are brought on the right side of the digital divide in order to put their experience into productive use.

One significant finding is that the grey digital divide is linked to poor healthcare in the present age. If seen from a general perspective, this connection gives rise to an important hypothesis: minimizing the grey digital divide is linked to an overall improvement in the health and welfare for the senior citizens. During present era of COVID-19 health emergency, most of the healthcare services are fast shifting towards digital platform. Trends indicate that the digital integration in healthcare practices eliminate unnecessary paperwork and queues at medical institutions. Many healthcare services can be availed over the Internet including appointment booking, secured communication with the medical practitioner, paying bills, and the list goes on. However, people on the wrong side of digital divide, especially elderly, will continue to suffer from effective healthcare in the near future. This is because electronic healthcare also known as eHealth care will dominate in the not-too-distant future but simultaneously ageing will also increase, as already warned by European Commission.

It must be remembered that elderly demand a reasonable period to get used to digital technologies which has to be respected by their tutors. In past, the trend in ICT education has been to deliver mostly theoretical knowledge with limited practicals which is not arguably enough for the people particularly in the old age. Elderly need their own time to learn new skills and this must be respected by their tutors with patience. Often elderly, will ask several questions and repeatedly so, to understand the basic ICT skills. Therefore, while training of the elderly is important, training of the tutors is equally significant by qualified ICT literates. This gives rise to the challenge of funding the training for elderly.

In developed countries, there are some efforts visible but these are still limited in scope. In the developing countries, situation is usually worse than the developed counterparts. This is mainly because money in poor countries must understandably go towards meeting pressing issues of basic human needs. Food and shelter still remains a far cry for millions of poor dwelling in the developing and underdeveloped countries. When portrayed in this way, it becomes self-explanatory that income disparity is a leading cause of the grey digital divide in the developing countries than their developed counterparts.

Mass media has brought us lot of information and same can be used to educate elderly. Video prompting mode of instruction, where videos are used as a means to education and provide skills holds a good promise to influence elderly regarding positive use of ICT. However, the question remains open on how to organize this ICT training to elderly worldwide.

In future, it is likely that the grey digital divide will be controlled to an extent. It is also likely that the digital divide in general will be controlled. However, there is no arguable reason to hypothesize that a full elimination of the grey digital divide or digital divide will ever be possible. While economies of scale will bring the prices of basic ICT equipment to control, the latest state-of-the-art technologies will continue to be expensive for most of the people. When seen from this perspective, the digital divide shall never cease to exist.

Admittedly, when current cohort of generation becomes older, they will be skilled to use ICT. However, the health challenges which come with old age shall continue to contribute to a continuing grey digital divide. Recent research has interrelated grey digital divide and mental health disorders. Although, the evidence is still fragmentary; it would tend to appear that digitally-excluded elderly are at a far higher risk of suffering mentally than their digitally-included peers. Elderly, who are not digitally-capable may not benefit from the state-of-the-art mental health treatment options available when there is already a global health emergency (COVID-19).

The findings indicate an important need for innovation-based research and efforts to bring the elderly into the digital sphere. It may be a difficult task for a single institution alone: a multidisciplinary research approach is recommended along academic, healthcare, and industrial discourses. It seems that the research topic of grey digital divide is seldom empirically investigated. As such further research is required preferably with fresh empirical approach on grey digital divide, that involves analysis of fresh primary data.

## Conclusions and Directions for Future

The present paper was designed to determine the current trends in the field of grey digital divide and trace any possible solutions to minimize the digital disparities among seniors. This review has provided us considerable evidence of a rising grey digital divide despite the apparent increase in diffusion of digital technologies across the globe. One of the more significant findings to emerge from this review is that the grey digital divide is compromising the effective healthcare of elderly. The grey digital divide is in all probability controllable to some extent but it cannot be conclusively ascertained yet, especially without a promising ecosystem of information literacy for seniors.

These findings have significant implications for the understanding of how solving the grey digital divide is essentially a multi-level challenge. Cross-collaboration research efforts among academic–healthcare–industrial discourses are required to design and innovate state-of-the-art digital inclusion initiatives for senior citizens. Not only elderly face troubles to learn ICT skills, but the trust on the information over Internet is gradually decreasing in the era of big data (ever-increasing accumulation of data). This further implies that tutors cannot use the same principles and techniques to deliver content to elderly as they do for young generation. Elderly take their own time to learn digital technologies, often need excessive patience, repeated reminders, slower learning, and sympathy of their tutors. The practical implication here is that tutors need to undergo specialized training to deliver digital education to elderly. Albeit, the cultural considerations across countries must also be fully acknowledged.

Although this review focuses on the grey digital divide, the findings may well have a bearing on solving the classic challenge of the digital divide. Digital technologies shall experience innovation and refutation over time, making way for the superior digital technologies. There shall remain a divide on the planet based on resources to access the latest state-of-the-art digital technologies. It is therefore likely that the digital divide, and consequently the grey digital divide shall never end and instead continue parallel to the continuity of new ICT devices and content. However, it can be controlled to an extent where it is not as big threat to economy as has been before.

What is now needed is a cross-national research involving fieldwork in the different countries of each continent. It would be worthwhile to learn how culture influences the digital divide, and the grey digital divide in particular. What we know so far based on recent research is that, among other reasons, elderly also refuse to learn (or they have no choice but to refuse) ICT due to various personal and cognitive factors such as lack of funds, lack of time, lack of learning ability, lack of willingness to learn, and frustration from their repeated error making during the learning process etc. Above all, we lack a system focused on engaging the elderly with the digital ecosystem. Large randomized controlled trials could provide more definitive evidence of the reasons behind continuing the grey digital divide. More broadly, research is also needed to determine emerging variants of digital divide with respect to the ever-changing ICT landscape.

As is the case with the review research, the articles selected for this paper may be limited in number as well as biased. To counter this limitation, a thorough filtering process was conducted carefully, to make sure that only relevant articles were selected according to the needs of the present paper. It could have been meaningful to conduct a research experiment involving samples of elderly for their views on digital inclusion; however, it fell outside the scope of the present paper in order to respect the advised national guidelines of maintaining social distance with respect to the on-going wave of COVID-19 pandemic.
